# Body Weight and Body Image

**DOI:** 10.1186/1472-6874-4-S1-S5

**Published:** 2004-08-25

**Authors:** Marion P Olmsted, Traci McFarlane

**Affiliations:** 1 Toronto General Hospital, University Health Network, 200 Elizabeth Street, Toronto, Canada, M5G 2C4; 2 Toronto General Hospital, University Health Network, 200 Elizabeth Street, Toronto, Canada, M5G 2C4

## Abstract

**Health Issue:**

Body weight is of physical and psychological importance to Canadian women; it is associated with health status, physical activity, body image, and self-esteem. Although the problems associated with overweight and obesity are indeed serious, there are also problems connected to being underweight. Weight prejudice and the dieting industry intensify body image concerns for Canadian women and can have a major negative impact on self-esteem.

**Key Findings:**

Women have lower BMIs than men, a lower incidence of being overweight and a higher incidence of being underweight. However, women across all weight categories are more dissatisfied with their bodies. Sixty percent of women are inactive, and women with a BMI of 27 or higher are more likely to be inactive than women with lower BMIs. The data show that women are aware of the health benefits of exercise, but there is a gap between knowledge and practice. When asked about barriers to health improvement, 39.7% of women cited lack of time and 39.2% lack of willpower.

**Data Gaps and Recommendations:**

Weight prejudice must be made unacceptable and positive body image should be encouraged and diversity valued. Health policies should encourage healthy eating and healthy activity. Health curricula for young students should include information about healthy eating, active lifestyle, and self-esteem. Physical activities that mothers can participate in with their families should be encouraged. Research should be funded to elucidate the most effective methods of getting women to become and remain physically active without focusing on appearance.

## Overview

Body weight is of both physical and psychological importance to Canadian women; it is associated with health status, physical activity, body image and self-evaluation. The body mass index (BMI) is the most common method of describing body weight standardized for height and is often used to derive "healthy" weights and to establish health risks. The Canadian standard for categorizing BMI is as follows: less than 20.0 underweight, 20.0 to 24.9 normal weight, 25.0 to 27.0 some excess weight, and more than 27.0 overweight. [1] International standards define obesity as a BMI of 30.0 or more. [2] Guidelines for body weight classification based on the World Health Organization recommendation to use BMI and waist circumference as indicators of health risk will soon be introduced. These new guidelines are not reflected in this report.

This section reviews some of the available evidence on body weight, body image and physical activity, and presents the results of an analysis of data from the National Population Health Survey (NPHS), 1996–1997.

### Body Weight and Health

Considerable attention has been focused on the association between body weight and health. An article published in Health Reports identified a number of chronic conditions that were associated with being overweight. [2] These included asthma, arthritis, back problems, high blood pressure, type 2 diabetes, thyroid problems, activity limitations, repetitive strain injuries and depression. For obese individuals, additional health risks included heart disease, urinary incontinence, ulcers and bowel disorders. In addition, the literature includes well-documented links between obesity and increased mortality and morbidity due to hypertension, dyslipidemia, diabetes mellitus, coronary heart disease, congestive heart failure, stroke, gallstones, osteoarthritis, sleep apnea, cancer (e.g. of the colon, breast, endometrium, gall bladder), menstrual abnormalities, impaired fertility and increased pregnancy risk. [3] There is no question that the risks associated with being overweight or obese are grave and need to be addressed in health reform policies (see the chapter in this report entitled "Physical Activity and Obesity). However, Ernsberger and Koletsky [4] argue that dieting behaviour may represent an alternative explanation for some of the negative consequences that have been linked historically with overweight and obesity. Also of interest are studies indicating that individuals who are overweight or obese are protected against certain conditions, including infectious diseases, chronic obstructive pulmonary disease, osteoporosis, mitral valve prolapse, intermittent claudication, renovascular hypertension, eclampsia, premature birth, anemia, type 1 diabetes, peptic ulcer, scoliosis and suicide. [5]

The relation between BMI and subsequent mortality is complex and varies markedly between different prospective epidemiologic studies. A quantitative meta-analysis of 23 major studies showed a U-shaped curve for both men and women, with increased risk of death when BMI was less than 23 or greater than 28. [6] In a study of 1.8 million Norwegians, it was estimated that 3.8% of deaths in women aged 30 to 79 could be attributed to obesity and 1.9% of deaths in the same age range could be attributed to underweight. [7] In another study, of Finnish women aged 25 to 64, the smallest and largest fifths of the population showed increased mortality; for women over 65, only being underweight increased the risk of death. [8] The negative health conditions associated with being underweight include ulcers and depression, [2] along with more complications and poorer prognosis associated with hypertension, diabetes and hyperlipidemia. [5] Women who develop eating disorders and drive their weight to dangerously low levels have additional problems. It is estimated that approximately 4% of Canadian women have a serious eating disorder, and as many as 10% of these women are expected to die from complications associated with their eating disorders. [9]

### Dieting

The view that some weights are unacceptable and that body weight is malleable has led to a large diet industry in North America, with estimated annual revenues of $35 to $50 billion. [10] Berzins outlined several commonly held misperceptions that contribute to the current cultural emphasis on weight control, including the incorrect beliefs that diets fail because of a lack of willpower and that being overweight is therefore the individual's fault. [10] The advertising industry promotes thinness as healthy and beautiful, and disproportionate attention is paid to the health risks of obesity while the risks associated with dieting are largely ignored. [4] A significant body of research has examined the effectiveness of various diet and exercise protocols in producing weight loss. Following an extensive review of this literature, Miller [11] reported that "each review article on the effectiveness of diet and exercise for weight control over the past 40 years concluded that diet and exercise are ineffective in producing substantial long-term weight loss for a majority of the participants." Given the evidence that weight reduction is an unattainable goal for most people, this may be a fruitless avenue for health promotion efforts.

### Body Image

Our current cultural preoccupation with thinness extends beyond the health risks associated with obesity. Nearly one half of North American women experience some degree of body image dissatisfaction. [12,13] In addition, women's body image has become poorer over the last three decades. [14] Although body dissatisfaction does occur in men, it is much more common in women, [15-17] and more pronounced in white than in Black women. [17] In a sample of 1,895 American women (51% Black), white women were more dissatisfied with both their size and overall appearance than Black women. [17]

Concern with body image and chronic dieting are so common that they are statistically "normal" for women; they also engender attitudes and behaviours that are self-defeating and self-destructive. [18] A significant number of women report putting career, romantic and social pursuits on hold until they lose weight. [19,20] For many women, self-esteem becomes tied to weight and shape, and negative feelings about the body generalize to the entire self. [21] This over-investment in thinness is a risk factor for the development of a serious eating disorder. [22] Women who are at particular risk include those with perfectionistic tendencies, those with very low self-esteem, and those who suffer from other psychiatric conditions such as anxiety or depression. [23] In addition, millions of Canadian women who never develop a full-blown eating disorder will squander time and psychological energy in the pursuit of thinness. The majority of them will be unsuccessful in this endeavour, will remain dissatisfied with their bodies, and will blame themselves.

### Weight Prejudice

Unfortunately, weight prejudice continues to be culturally condoned in our society. One particularly destructive mode of transmission is through teasing. Although girls with a higher BMI are more likely to be teased, body dissatisfaction is more strongly predicted by teasing than by BMI. [24] Body dissatisfaction is also more strongly correlated with low self-esteem than with BMI in adolescent girls. [25] Although evidence related to the causal order of the relation between self-esteem and body dissatisfaction is limited and the two likely have a reciprocal influence, self-esteem and self-definition may incorporate a vulnerability to the socio-cultural message. In this regard, Sherwood and Neumark-Sztainer [26] have shown that 10- and 11-year-old girls who were considered dieters had greater internalization of the socio-cultural ideal than girls who were not dieters, even though both groups had similar exposure to teen magazines. Additional research is needed to clarify the mechanisms by which weight prejudice and the idealization of thinness are selectively internalized.

### Physical Activity

A large body of literature provides evidence that moderate physical activity has both physical and mental health benefits, including stress reduction and the prevention of heart disease, cancer, diabetes, osteoporosis and depression. [27-29] The results of a recent article published in Health Reports indicate that regular and at least moderate physical activity is associated with reduced odds of heart disease and depression. [30] Miller [31] has suggested that some of the morbidity and mortality attributed to obesity in research studies may be the result of inactivity rather than BMI or fatness. Increased physical activity results in improved health at all body weights, including obesity. [31] In Canada, 2.5% of the total direct health care costs for 1999 were attributed to physical inactivity. [32]

More men than women engage in regular exercise and, among women, appearance and weight control are cited as more important motivators than physical fitness. [33,34] Furthermore, 75% of fitness articles in popular women's magazines encourage readers to exercise to be more attractive, whereas only 40% focus on improved health or well-being. [35]

## Method

### Data

Cross-sectional data from the National Population Health Survey (NPHS) were analyzed. The NPHS is a national survey that collects information every two years (see Appendix A for details about the methods of data collection for the NPHS). The analyses included male and female respondents aged 20 to 64, and excluded women who were pregnant.

Tables 2 (BMI and geographic region) and 3 (BMI and ethnicity) have been age-standardized to the 1991 Canadian population for the population between 20 and 64. Five-year age groups were used to calculate the age-standardized estimate.

### Measures

Body Mass Index (BMI): BMI is calculated by dividing the weight in kilograms by height in metres squared.

#### Body Dissatisfaction

Respondents were asked to state a weight that they would consider to be their ideal weight. The discrepancy between their actual weight and their ideal weight was used as an indicator of body dissatisfaction. In addition, respondents were asked to nominate a description of their current weight. Descriptions included: just right, overweight and underweight.

#### Physical Activity Index

In the NPHS, participants were asked to list their leisure-time physical activities for the previous three months. There were also questions on frequency of participation and amount of time per occasion. Based on independently established values for the energy demands of each activity, an index of total kilocalorie expenditure was calculated. Level of activity was classified according to estimated kilocalories per kilogram of body weight per day: active (3.0 kcal/kg/day or more), moderate (1.5–2.9 kcal/kg/day) or inactive (less than 1.5 kcal/kg/day).

#### Barriers to Health Improvement

Respondents were asked to nominate factors that interfered with their ability to participate in health improvement activities. Factors included: lack of time, lack of willpower, disability/health problems, too tired, too costly, too stressed, too difficult and other.

## Results

### Sex Differences

**Figure 1:**
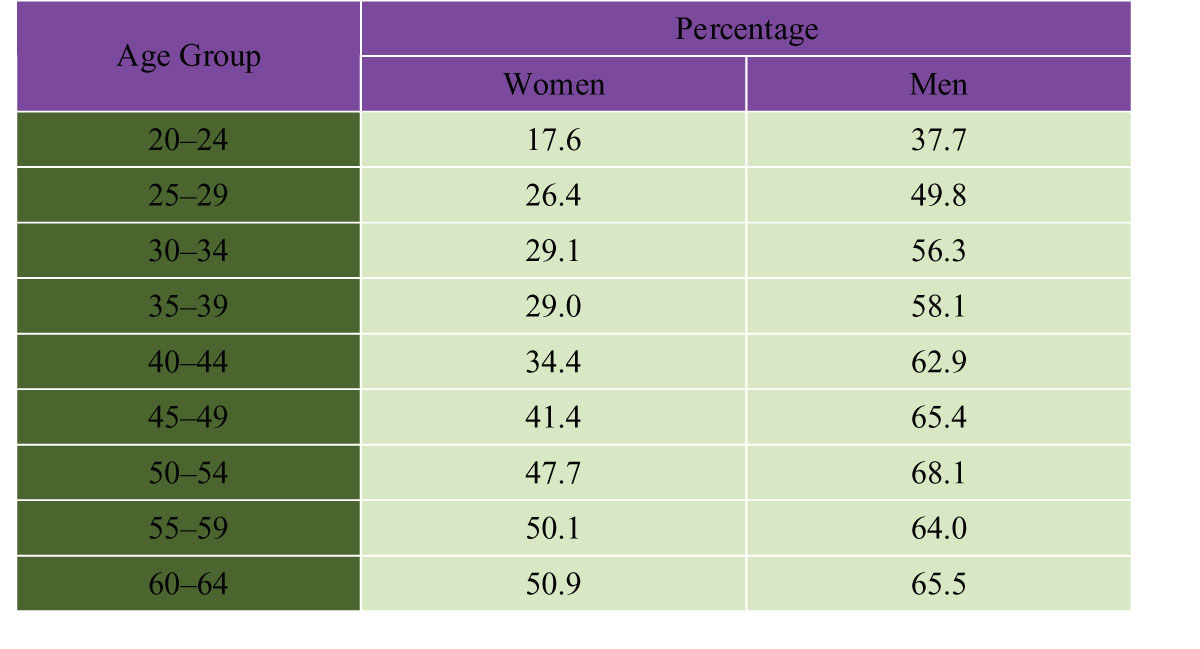
**Percentage of Canadian Women and Men with a BMI Greater than or Equal to 25 in Each Age Category **Source: Statistics Canada, NPHS 1996–1997

On average, women have lower BMIs than men. Data from the National Population Health Survey show that BMI increases with age in Canadian women and men (see Figure 1). By the age of 55, half of Canadian women are in the "some excess weight" or "overweight" categories. In contrast, half of Canadian men are in these weight categories by age 25. Averaged across age groups, 12.1% of women and 22.6% of men are in the "some excess weight" category, and 23.4% (Confidence Interval [CI] 22.3, 24.6) of women and 34.4% (CI 33.1, 35.7) of men are considered "overweight"; 50.5% of women and 40.2% of men are in the "normal weight" category, and 14.0% (CI 13.0, 15.0) of women and 2.8% of men (CI 2.3, 3.2) are "underweight."

The emphasis on overweight as a health problem appears to match the demographic profile of Canadian men more strongly than that of Canadian women. Among women, the incidence of overweight is significantly lower, and the incidence of underweight is significantly higher. This suggests that a singular focus on excess weight is too simplistic. Furthermore, the emphasis on weight reduction contributes to a major health concern for Canadian women.

**Figure 2:**
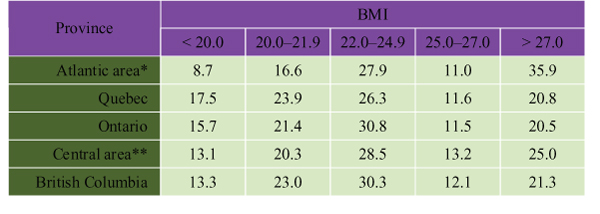
**Percentage of Canadian Women from Each Province in Each BMI Category **Source: Statistics Canada, NPHS, 1996–1997

**Figure 3:**
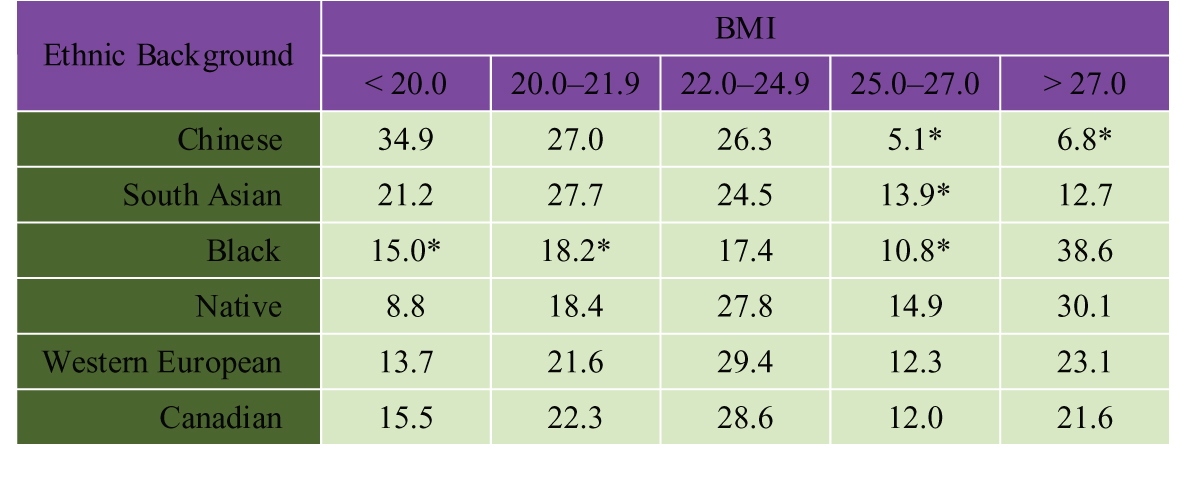
**Percentage of Canadian Women in Each BMI Category as a Function of Ethnic Background **Source: Statistics Canada, NPHS, 1996–1997

BMI is presented by geographic region and ethnic background in Figures 2 and 3. These data are age-standardized to the 1991 population; however, some of the original cells contained cell counts less than 30 and have been noted. For women who live in Quebec, Ontario, British Columbia and the central provinces the prevalence of "overweight" is lower and the prevalence of "underweight" is higher than that for women living in the Atlantic provinces (see Figure 2). "Overweight" appears to be more common in women from Black and Native groups, while "underweight" is more common among Chinese and South Asian groups (see 3). Some of these differences may be artifacts of the measurement system that was applied, as BMI does not differentiate between "fatness" and size (see limitations section).

### Body Dissatisfaction

Sex and ethnic differences in body dissatisfaction indicate that it is an aspect of self-definition that supersedes actual body weight. Data from the NPHS show that 81% of Canadian women with a BMI less than 20 (i.e. underweight) considered themselves "just right" and 2% felt they were "overweight." Women with a BMI between 20 and 22 (i.e. below average but "acceptable") reported a mean ideal weight that was 3 kg less than their current weight. This is in contrast to men in the same BMI range who needed to gain almost 7 kg to reach their preferred weight. Although the incidence of overweight is higher among men, it is women who are more dissatisfied with their bodies, and this dissatisfaction occurs across all weight categories (see the chapter in this report entitled "Eating Disorders" for more information about body image).

### Physical Activity

**Figure 4:**
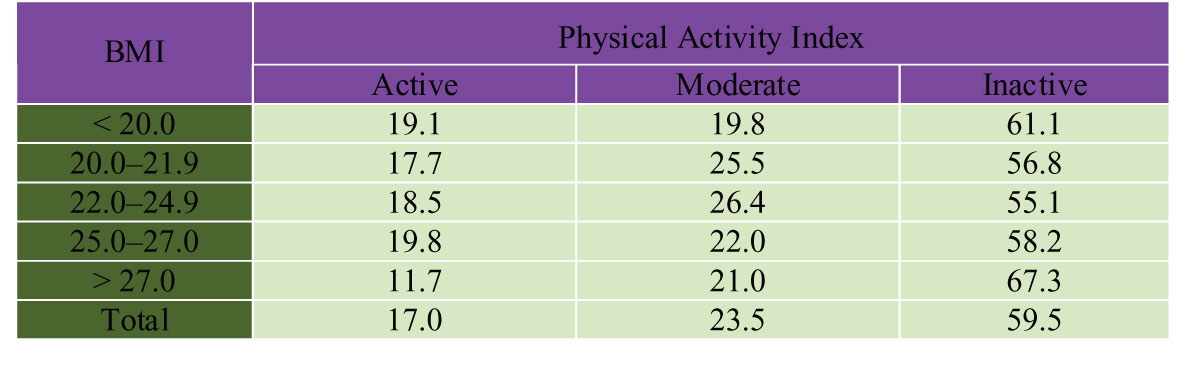
**Percentage of Canadian Women in Each Physical Activity Category for Each BMI Group **Source: Statistics Canada, NPHS, 1996–1997

**Figure 5:**
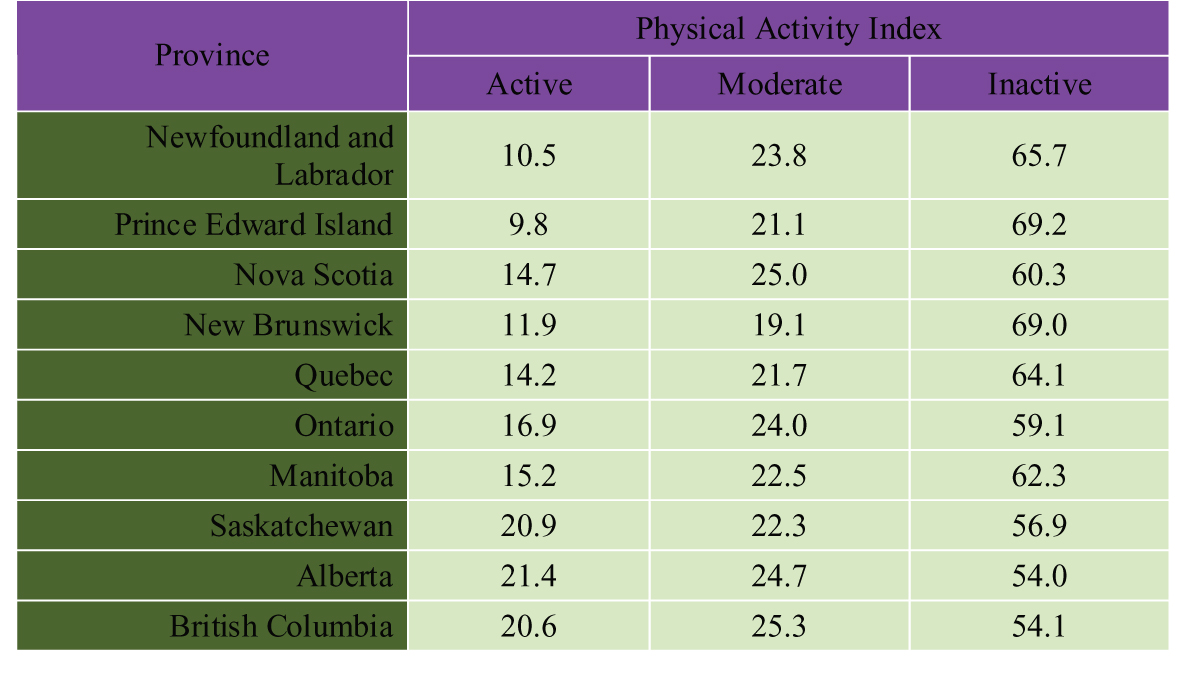
**Percentage of Canadian Women from Each Province in Each Physical Activity Category **Source: Statistics Canada, NPHS, 1996–1997

Data from the NPHS, based on the Physical Activity Index, show that 57.6% of Canadian men and 59.5% of Canadian women are classified as inactive, and 20.0% of men and 17.0% of women are classified as active. Women with a BMI of 27 or greater are more likely to be inactive than women with lower BMIs (see Figure 4). There were also differences across geographic regions and ethnic groups. Being inactive was less common among women who live in Alberta and British Columbia and more common among women who live in Prince Edward Island, Newfoundland and Labrador, and New Brunswick (see Figure 5).

A majority of Canadian women in the NPHS reported that they believed they should improve their health, and in all BMI categories increased exercise was endorsed as the top priority for health improvement. Thus, Canadian women do seem to be aware of the health benefits of exercise, but there is a gap between knowledge and practice.

### Barriers to Health Improvement

**Figure 6:**
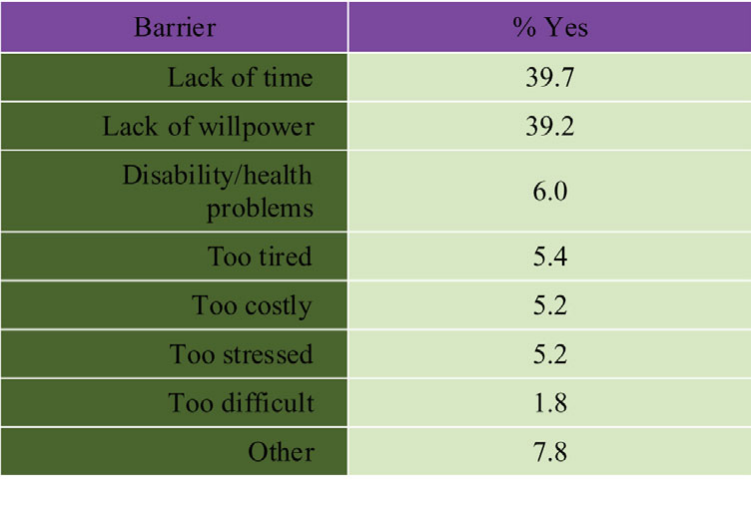
**Barriers to Health Improvement Reported by Canadian Women **Source: Statistics Canada, NPHS, 1996–1997

When asked about barriers to health improvement, 39.7% of Canadian women in the NPHS cited lack of time and 39.2% cited lack of willpower; other obstacles were cited much less frequently (see Figure 6).

Nominating lack of willpower as a significant problem is self-blaming and self-defeating, as there is no clear way to change the situation. In addition, Chen and Millar [36] have noted that women who are overweight or have children under age 18 are less likely than other women to engage in moderate leisure-time activity, whereas these factors have no influence on men. Similarly, Whiteley and Winett [37] observed that child-care and home-care responsibilities are barriers to fitness for women, as are impossible-to-attain body image ideals. It is not surprising that women feel pressed for time and have difficulty making personal activity take priority over other demands. However, to the extent that weight, self-blame for being weak-willed, and unrealistic body image goals prevent women from being active, the cultural paradox is complete: in a society that claims to value fitness and thinness as measures of health, women who are not fit and thin are discouraged from participating in health improvement.

## Discussion

### Data Limitations

The National Population Health Survey (NPHS) data are subject to the problems inherent in self-reporting. There was no objective measurement of height and weight. In a recent study it was determined that individuals consistently overestimate their height, and, for those without an eating disorder, underestimate their weight. [38] Therefore the data presented in this report are likely to be an underestimate of overweight and obesity. Other studies have concluded that self-reported data tend to underestimate the prevalence of overweight and obesity by approximately 10%. [39,40]

In addition, the body mass index (BMI) is useful for only a general analysis of weight categories and their relationship to health. Although BMI is correlated with body fat it is not a perfect measurement. For example, an athlete may have a BMI of 31 but be very muscular and lean. It would be inaccurate and misleading to consider this individual obese. Also, it is important to note that BMI is based on the same average body composition for men and women; however, men are generally more muscular than women. Therefore some of the men in the "some excess weight" category may not have excess body fat (but high muscle) and some of the women at the higher end of the low-risk category may have a higher body fat than their BMI suggests. BMI is more valuable when used in conjunction with a Waist-to-Hip Circumference ratio or a waist circumference measurement. [41] These circumference variables were not collected by the NPHS, but are reflected in the new guidelines for body weight classification.

The measurement of body dissatisfaction used in this study is not a direct measurement of dissatisfaction. Rather, it is inferred that those who consider themselves overweight or obese, or who report an ideal weight that is lower than their objective weight, are dissatisfied with their bodies. This assumption, of course, may not be true. Many of these respondents may in fact be satisfied with their bodies and similarly many respondents who report feeling "just right" or who are at their ideal weight may be dissatisfied with their bodies. There is also no way to determine the intensity of the body dissatisfaction, the level of preoccupation, and the degree to which the dissatisfaction interferes with the respondents' lives. A direct measure assessing body dissatisfaction and level of interference was not included in the NPHS.

## Recommendations

### Policy Implications and Recommendations

1. Weight prejudice must be made unacceptable. Initiatives should focus on raising awareness in the general public, but schools and primary care physicians may be particularly good avenues of dissemination.

2. Positive body image should be encouraged and diversity valued, as in the approach taken to promote multiculturalism.

3. Body image disparagement, chronic dieting and exercise to improve appearance need to be acknowledged as vehicles of oppression of women. Policies should encourage all Canadians to take pride in developing a healthy lifestyle with a focus on healthy eating (i.e. not too much or too little) and healthy activity every day. The emphasis should be on healthy living and not on looking more attractive.

4. Similar to what is planned in Alberta, health curricula for students starting as early as grade 2 should include information about healthy eating, an active lifestyle and self-esteem. Students can be taught to view the media's obsession with thinness with a critical eye, to understand that negative comments about the body are a form of harassment, and to derive self-worth from areas other than appearance. [42]

5. Physical activities that mothers can participate in with their families should be encouraged as one method of addressing competing demands and limited time.

6. Research should be funded to elucidate the most effective methods of getting women to become and remain physically active without focusing on weight control or appearance.

## Note

* The views expressed in this report do not necessarily represent the views of the Canadian Population Health Initiative, the Canadian Institute for Health Information or Health Canada.

Body Mass Index

BMI is calculated by dividing the weight (in kilograms) by height (in metres) squared. For example, to calculate the BMI of someone 5 feet 4 inches tall, weighing 145 pounds, it is first necessary to convert that person's height into metres (64 inches × 2.54 = 162.5 centimetres or 1.625 metres) and weight into kilograms (145 pounds × .454 = 65.8 kilograms). The BMI of this individual is 24.9, a result of dividing weight (65.8 kilograms) by height, squared (1.625 × 1.625 = 2.64 sq. metres). According to the Canadian standards this individual would be considered to be normal weight.
